# What is hot in experimental thyroidology?

**DOI:** 10.1186/1756-6614-4-S1-I1

**Published:** 2011-08-03

**Authors:** Heike Biebermann

**Affiliations:** 1 Institute of Experimental Pediatric Endocrinology, Charité Universitätsmedizin Berlin, Augustenburger Platz 1, 13353 Berlin, Germany

## 

Over the recent years, considerable progress has been made in elucidating processes that are critical for thyroid hormone (TH) synthesis and action. In this issue of Thyroid Research several German groups dedicated to thyroid research have assembled and discussed novel aspects of thyroid hormone action with the perspective to improve diagnosis and therapy of thyroid disorders by better understanding disturbed thyroid differentiation. The over-arching theme addressed in the contributions of this special issue relates to the main focus of current thyroid research and treatment of thyroid disesase, i.e. “What defines thyroid health?” [1]. Thereby the putative role of seemingly established players in TH synthesis and action are revisited.

A report on a patient characterized by a novel germline TSH-receptor mutation links this case of non-autoimmune hyperthyroidism to new impliations in TSH-receptor structure [2]. The study highlights the importance of introducing novel diagnostic approaches to the clinics, because this particular mutation in the TSH-receptor is correlated to altered G_q_-signalling pathways, thereby pointing to an important role of Gq signalling in the thyroid

It is known only since recently that transport of TH is an active and regulated mechanism. The monocarboxylate transporter 8 (MCT8) is a crucially important transporter of TH and it transports triiodothyronine (T3) specifically well. Mutations in MCT8 are known to cause a severe syndrome of X-linked mental retardation known as Allan-Herndon Dudley syndrome. Based on homology modeling all of the currently reported pathogenic mutations in MCT8 are studied in the article by Kleinau et al. [3]. A focus is set on identification of those MCT8 mutations that are most sensitive for substrate transport with the goal to simulate *in silico* the potencies of future therapies based on TH transporter-specific inhibitors mimicking TH structurally.

Besides MCT8 other transporters have been proposed to play important roles in TH transport (MCT10, OATP1C1, OATP1A2, OATP14, LAT1 and LAT2). The structure-function relationships of primary TH transporters like MCT8 and additional, so-called secondary TH transporters such as those listed above is reviewed by Kinne et al. [4] with special attention on their expression profiles, transport specificities and substrates. Similarities and differences in structure and molecular features of the known and suspected TH transporters are compared between man and mouse, in particular asking their crucial impact on TH actions in the central nervous system.

TH actions in the brain and clinical consequences of non-classical, rather novel treatment protocols to relieve symptoms of severe mood and depression disorders is reviewed in the article by Pilhatsch et al. [5].

For a long time, only genomic actions of TH, namely of T3 were recognized by both, thyroid researchers and clinicians. However this picture has to be revised as non-classical, non-genomic TH actions were described which lead to activation of alternative signaling pathways via PI3K and MAPK. The impact and perspectives of these recent findings are summarized by Möller and Bröcker-Preuß [6] who comment on the outcomes of activation of alternative signaling pathways on transcriptional regulation of TH target cells.

Non-classical and classical TH actions must be taken into consideration for better management of thyroid malignancies. Moreover, it is increasingly appreciated that disturbances in TH synthesis occur in thyroid tumorigenesis and that disturbed TH action affects carcinogenesis in general. Of the many novel aspects in thyroid carcinogenesis, it was the recent identification of miRNAs and their intricate involvement in the regulation of transcriptional, translational and epigentic control mechanisms that provides one of the most promising diagnostic and high-potential, future therapeutic tool. The aspects of miRNA relevance for thyroid pathologies are discussed by Braun and Hüttelmaier [7].

Likewise, regulation of transcription and translation by RNA binding proteins is addressed in the article by Trojanowicz et al. The adenylate uridylate-rich element binding proteins AUF together with HuR was reported to regulate mRNA stability and could hence participate in thyroid carcinogenesis. RNA binding proteins may harbor multifunctional properties that not only affect thyrocyte differentiation but could also affect TH synthesis [8].

Finally, the characterization of protein transport pathways and the identification of altered trafficking in thyroid carcinoma cells are approached in the experimental study by Tedelind et al. [9] that investigated the cathepsins which are known as thyroglobulin-processing enzymes with important functions in TH biosynthesis.

We believe that the papers collected for this special issue of Thyroid Research will have a stimulatory effect not only for thyroid researchers but will be interesting for those from associated disciplines.

## Competing interests

The author confirms that there are no competing interests.
